# Atypical histone targets of PHD fingers

**DOI:** 10.1016/j.jbc.2023.104601

**Published:** 2023-03-11

**Authors:** Joshua C. Black, Tatiana G. Kutateladze

**Affiliations:** Department of Pharmacology, University of Colorado School of Medicine, Aurora, Colorado, USA

**Keywords:** PHD, histone, binding, chromatin, inhibitor

## Abstract

Plant homeodomain (PHD) fingers are structurally conserved zinc fingers that selectively bind unmodified or methylated at lysine 4 histone H3 tails. This binding stabilizes transcription factors and chromatin-modifying proteins at specific genomic sites, which is required for vital cellular processes, including gene expression and DNA repair. Several PHD fingers have recently been shown to recognize other regions of H3 or histone H4. In this review, we detail molecular mechanisms and structural features of the noncanonical histone recognition, discuss biological implications of the atypical interactions, highlight therapeutic potential of PHD fingers, and compare inhibition strategies.

A large family of proteins that mediate fundamental cellular processes in the nucleus contain a plant homeodomain (PHD) finger (1, 2). This domain is characterized by a canonical Cys_4_HisCys_3_ motif (or less common Cys_4_HisCys_2_His), which coordinates two zinc ions and can be present as a single module or in multiple copies in the same protein. The PHD finger was originally discovered in the *Arabidopsis* protein HAT3.1 in 1993 (3), but its biological role remained unclear until 2006 when PHD fingers of the proteins BPTF and ING2 were shown to bind or ‘read’ the posttranslational modification (the so called epigenetic mark), trimethylated lysine 4 of histone H3 (H3K4me3) (4, 5, 6, 7). A year later, a second major subset of PHD fingers selective for the unmodified H3 tail were identified (8, 9). Since then, several dozen PHD fingers capable of associating with the amino-terminal tail of histone H3, either methylated or unmodified, have been discovered and structurally and functionally characterized. These two subsets of typical PHD fingers constitute an established and well-characterized family of epigenetic readers (10, 11, 12).

The binding of typical PHD fingers to the uttermost N-terminus of H3 stabilizes or recruits scaffolding and enzymatic host proteins and their complexes to specific genomic sites and therefore is vital to many DNA-templated processes, including gene transcription and DNA replication, recombination, and repair (13, 14, 15). Transcriptional regulation is the most common function of the proteins containing typical PHD fingers, whose histone binding can promote transcriptional activation or repression. The typical PHD finger–containing proteins are directly implicated in chromatin remodeling, nucleosome sliding, and DNA damage repair. Various nuclear signaling pathways that mediate the cell cycle, growth, and differentiation require the histone-binding function of typical PHD fingers, whereas aberrations in PHD fingers are associated with pathological developments.

Although diverse and sometimes opposing cellular processes involve methylated and unmodified states of the histone H3 tail, there are more similarities than differences in the mechanisms by which PHD fingers recognize these ligands. In the complexes of PHD fingers with either H3K4me3 or unmodified H3 tail, the histone ligand occupies the same extended and primarily negatively charged–binding site (Fig. 1*A*). The H3 tail forms the third antiparallel β-strand (typically longer in the case of unmodified H3), pairing with the existing double-stranded β-sheet of the PHD finger. The positively charged amino group of A1 of the H3 tail is hydrogen bonded to one or more backbone carbonyls of the PHD finger, whereas R2 of H3 lays in a negatively charged groove where its guanidinium moiety is restrained through hydrogen bonds and ionic interactions (Fig. 1, *B* and *C*). The differences arise in the recognition of methylated or unmodified K4 of H3. The trimethylated K4 is bound in a binding pocket consisting of one to four aromatic residues and named the ‘aromatic cage’. The aromatic rings and K4me3 are engaged in cation-π, hydrophobic, and van der Waals interactions. In contrast, those PHD fingers that bind to unmodified H3 lack the aromatic cage and instead use a set of negatively charged residues to constrain the ammonium group of K4 *via* hydrogen bonds and salt bridges. Such a difference in the mode of K4 *versus* K4me3 recognition is critical for biological activities—PHD fingers specific toward H3K4me3 usually do not bind unmodified H3 tail, and vice versa, those that bind unmodified H3 tail do not bind H3K4me3. The lower di- and mono-methylation states of K4 impede binding of either subset of PHD fingers, leading to a decrease of binding affinities from low micromolar to millimolar.

**Figure 1:**
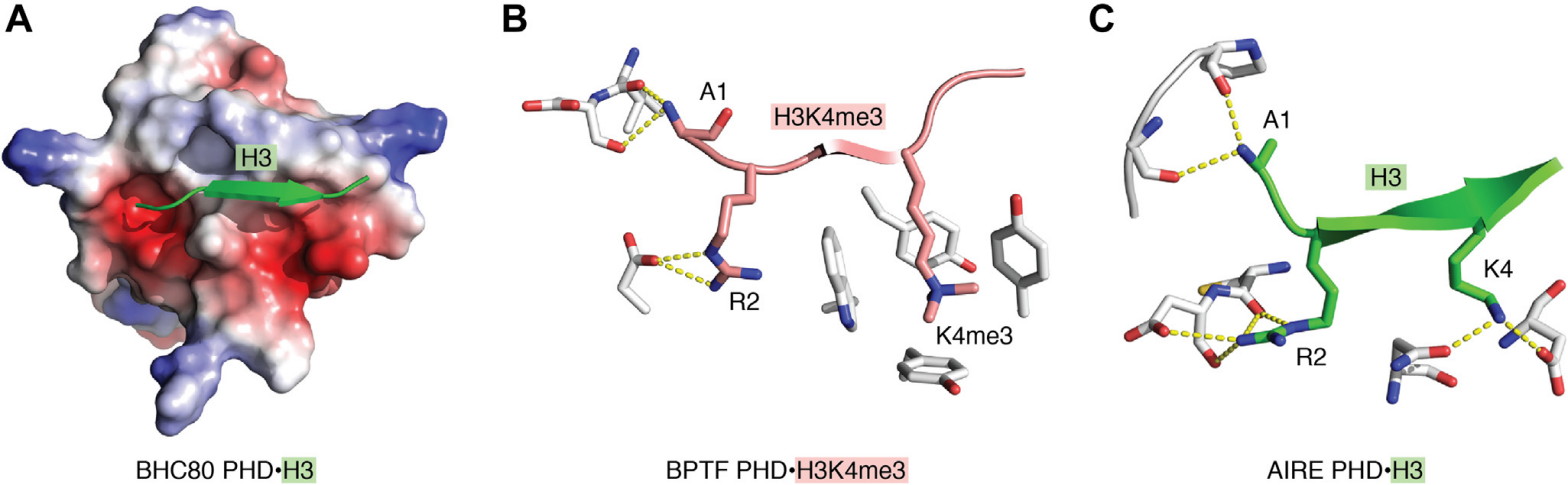
**Canonical histone H3-binding mechanism of PHD fingers.***A*, the crystal structure of the BHC80 PHD finger in complex with the unmodified histone H3 peptide (PDB ID 2PUY). Electrostatic surface potential of the PHD finger is shown with *blue* and *red colors* representing surface positive and negative charges, respectively. The H3 peptide is depicted as *green ribbon*. *B*, a ribbon diagram of the H3K4me3-binding site derived from the crystal structure of the BPTF PHD finger (*gray*) in complex with the H3K4me3 peptide (*dark pink*) (PDB ID 2F6J). Intermolecular hydrogen bonds are indicated by *yellow dash lines*. The H3K4me3 peptide residues are shown as *sticks* and labeled. *C*, a ribbon diagram of the unmodified H3-binding site derived from the crystal structure of the AIRE PHD finger (*gray*) in complex with the H3 peptide (*green*) (PDB ID 2KE1). Intermolecular hydrogen bonds are indicated by *yellow dash lines*. The H3 peptide residues are shown as *sticks* and labeled. PHD, plant homeodomain.

In addition to being readers of the amino terminus of histone H3, several PHD fingers have been reported to associate with other regions of H3 or histone H4. In this review, we compare the canonical and noncanonical histone-binding mechanisms of PHD fingers and discuss biological implications of the atypical interactions with histones.

## Recognition of H4K16ac

**Figure 2:**
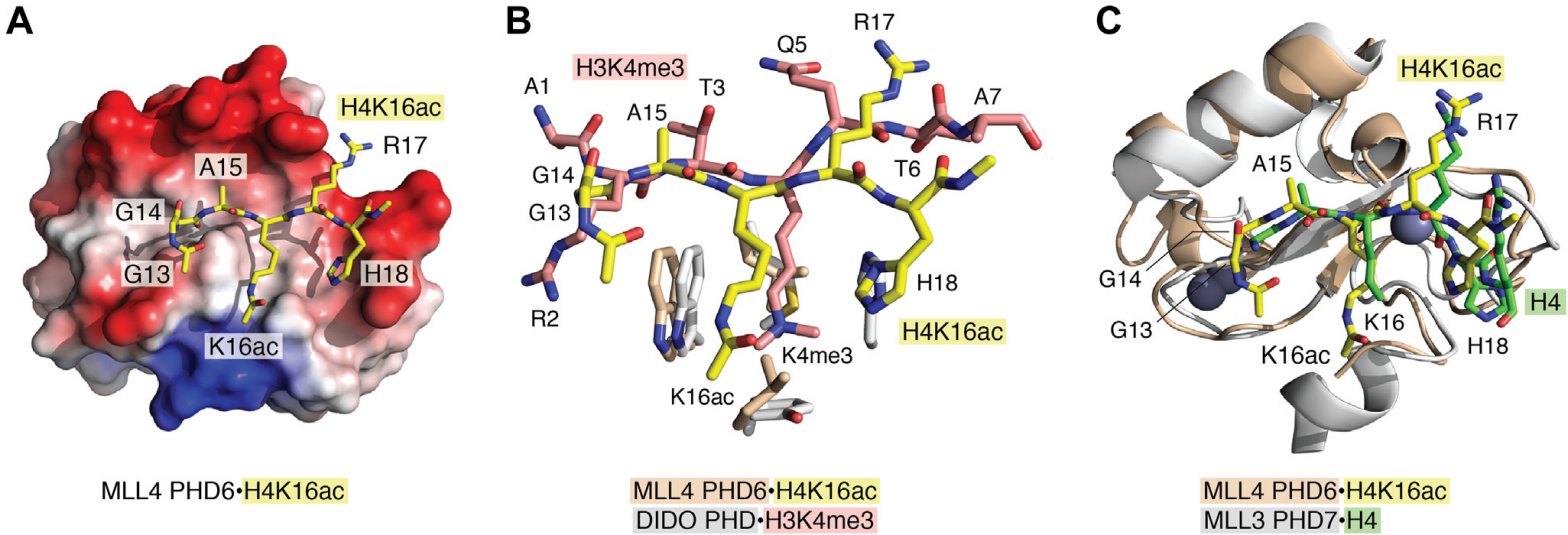
**Structural basis for the recognition of H4 by PHD fingers.***A*, the solution NMR structure of the MLL4 PHD6 finger in complex with H4K16ac peptide (PDB ID 6O7G). Electrostatic surface potential of the PHD6 finger is shown with *blue* and *red colors* representing surface positive and negative charges, respectively. The H4K16ac peptide is depicted as *yellow sticks*. *B*, overlay of the H4K16ac-binding site derived from the structure of the MLL4 PHD6 finger (*wheat*) in complex with H4K16ac peptide (*yellow*) (PDB ID 6O7G) and the H3K4me3-binding site derived from the structure of the DIDO PHD finger (*gray*) in complex with H3K4me3 peptide (*dark pink*) (PDB ID 4L7X). The K4me3-aromatic cage residues and the K16ac-hydrophobic groove residues are shown as *sticks*. Histone peptides residues are labeled. *C*, structural overlay of the MLL4 PHD6 finger (*wheat*) in complex with H4K16ac peptide (*yellow*) (PDB ID 6O7G) and the MLL3 PHD7 finger (*gray*) in complex with unmodified H4 peptide (*green*) (PDB ID 6MLC). MLL, mixed lineage leukemia, PHD, plant homeodomain.

The sixth PHD (PHD6) finger of mixed lineage leukemia 4 (MLL4/KMT2D) and the seventh PHD (PHD7) finger of MLL3 (KMT2C) have been found to bind H4K16ac (acetylated lysine 16 of H4) (16) rather than modified or unmodified H3 (17). The solution NMR structure of the H4K16ac-bound PHD6 finger of MLL4 shows that the H4K16ac peptide occupies the same histone H3-binding site and, similarly to the H3 tail, forms a third antiparallel β strand paring with the β-sheet of PHD6 (16) (Fig. 2*A*). Structural overlay of the bound H4K16ac peptide and bound H3K4me3 peptide from unrelated PHD complexes reveals unpredictably similar conformations of not only the peptides’ backbones but also the residues’ side chains (Fig. 2*B*). The residues T3, K4me3, and Q5 of H3K4me3 superimpose well with the residues A15, K16ac, and R17 of H4K16ac. The neutral side chain of K16ac is bound in the hydrophobic groove, consisting of tryptophan and two leucine residues of the PHD6 finger, and again, the side chains of these hydrophobic residues overlay very well with the side chains of the residues that form the aromatic cage for H3K4me3 (Fig. 2*B*). Orientation of the aromatic rings of tryptophan that creates one of the walls in the acetyllysine-binding groove in particular is almost identical to the orientation of the aromatic rings of invariable tryptophan that forms a wall in the trimethyllysine-binding cage.

Notably, the surface charge distribution in the MLL4 PHD6 finger differs from that of a typical H3-recognizing PHD finger, most likely accounting for the distinct histone selectivity (compare Figs 1*A* and 2*A*). The typical PHD finger has highly negatively charged pockets that accommodate NH_3_^+^ of A1 and the positively charged guanidino group of R2 (and the positively charged ammonium group of K4 in the case of unmodified H3) but a neutral surface on another side of the bound peptide. In contrast, an opposite surface charge distribution is seen in the PHD6 finger of MLL4, with the H4K16ac peptide being bound in a primarily neutral-binding site which is enclosed by a highly negatively charged wall where R17 is bound (16).

Another unique mechanistic feature of the H4K16ac recognition is that the MLL4 PHD6 finger does not require the free amino-terminal NH_3_^+^ group for the interaction and instead binds to the middle part of histone H4 tail. The flexible G13-G14 sequence in H4 ensures proper fitting of the N-terminal part of the H4K16ac peptide in the binding cavity of the PHD finger, where A1 of H3 is usually bound. G13 of H4 forms a sharp turn, allowing G14 to occupy this cavity and avoid steric clashes. This binding mode is corroborated by the crystal structure of the PHD7 finger from homologous MLL3 in complex with unmodified H4 (18) (Fig. 2*C*). Finally, the importance of acetylation of H4K16 for the tight interaction with the MLL4 PHD6 finger was demonstrated through measuring binding affinities. Acetylation of H4K16 enhances binding to the unmodified H4 peptide ∼13 fold, but acetylation of H4K12 or H4K20 does not augment the binding activity (16). Importantly, the finding that some PHD fingers can recognize H4K16ac has expanded the family of acyl-lysine–specific epigenetic readers with unique binding modes (19, 20, 21, 22, 23).

## Functional significance of binding to H4K16ac

MLL4 is a member of the KMT2 family of methyltransferases and a catalytic subunit of the multicomponent COMPASS-like complex that regulates enhancer elements (24). Mammalian enhancers are normally marked by elevated levels of H3K4me1 (monomethylated lysine 4 of H3) deposited by the methyltransferases MLL3 and MLL4 and H3K27ac (acetylated lysine 27 of H3) deposited by the acetyltransferases CBP/p300 (25, 26, 27). However, a subset of CBP/p300- and H3K27ac-independent enhancers in mouse embryonic stem cells was shown to rely instead on H4K16ac and H3K4me1 (28). Recent genetic studies demonstrate that MLL4 colocalizes with H4K16ac almost exclusively on active enhancers and active promoters of target genes in mouse preadipocytes, and this co-occupancy depends on and requires the intact PHD6 finger and H4K16-acetyltransferase activity of MOF (16). It will be interesting to explore whether recognition of H4K16ac by the MLL4 PHD6 finger could provide a tool for delineating the mechanisms by which H4K16ac- or H3K27ac-enriched enhancers control expression and functions of genes.

MLL4 is one of the most frequently altered genes in cancer (29, 30). Many of these alterations are frameshifts that yield truncated MLL4 proteins retaining the PHD fingers but lacking the C-terminal catalytic domain and thus are defective in methylation (cBioPortal). Some residues located in or near the H4K16ac-binding site in the PHD6 finger are also found mutated in cancer samples. These include missense mutations of E1516, E1517, E1540, E1544, and D1548. In addition, mutations of G13, G14, A15, and R17 in the H4 tail region that associates with PHD6 have also been reported (31). These observations point to the importance of the PHD6-H4K16ac interfacial residues in function of MLL4 and encourage further investigation into the role of impaired H4K16ac binding in cellular processes and diseases.

## Binding of PHD to H3G34R

**Figure 3:**
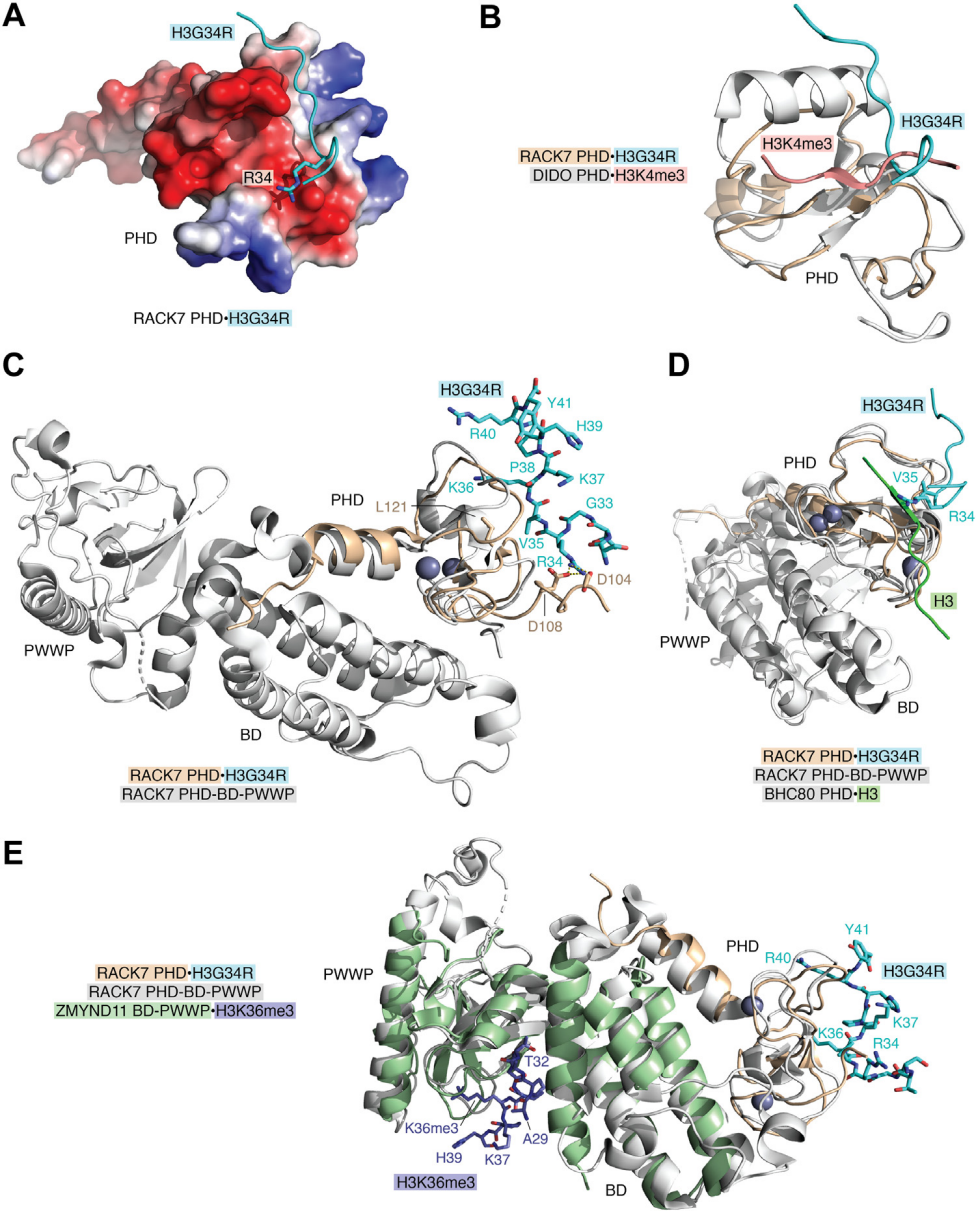
**Structural basis for the recognition of H3G34R.***A*, the crystal structure of the RACK7 PHD finger in complex with H3G34R peptide (PDB ID: 7CWH). Electrostatic surface potential of the PHD finger is shown with *blue* and *red colors* representing surface positive and negative charges, respectively. The H3G34R peptide is depicted as a *cyan ribbon*, with R34 shown as *sticks* and labeled. *B*, overlay of the crystal structures of the RACK7 PHD finger (*wheat*) in complex with H3G34R peptide (*cyan*) (PDB ID: 7CWH) and the DIDO PHD finger (*gray*) in complex with H3K4me3 peptide (*dark pink*) (PDB ID 4L7X). Histone peptides are depicted as *ribbons*. *C*, structural overlay of the RACK7 PHD finger (*wheat*) in complex with H3G34R peptide (*cyan*) (PDB ID: 7CWH) and the RACK7 PHD-BD-PWWP cassette (*gray*) (PDB ID 5B73). The H3G34R peptide residues and the PHD finger residues involved in the interaction with the peptide are shown as *sticks* and labeled. Intermolecular hydrogen bonds are indicated by *yellow dash lines*. The zinc ions are *gray spheres*. *D*, structural overlay of the RACK7 PHD finger (*wheat*) in complex with H3G34R peptide (*cyan*) (PDB ID: 7CWH), the RACK7 PHD-BD-PWWP cassette (*gray*) (PDB ID 5B73), and the unmodified H3 (*green*)-bound BHC80 PHD finger (*gray*) (PDB ID 2PUY). H3G34R peptide and unmodified H3 peptide are shown as ribbons, and R34 and V35 of the oncohistone are labeled. *E*, structural overlay of the RACK7 PHD finger (*wheat*) in complex with H3G34R peptide (*cyan*) (PDB ID: 7CWH), the RACK7 PHD-BD-PWWP cassette (*gray*) (PDB ID 5B73), and the ZMYND11 BD-PWWP cassette in complex with H3K36me3 peptide (*dark blue*) (PDB ID 4N4H). PHD, plant homeodomain.

The PHD finger of RACK7 (receptor for activated C-kinase, also known as ZMYND8) has been identified as a reader of oncogenic histone H3.3G34R (H3G34R for simplicity here), but this PHD finger is also capable of binding to the unmodified H3 tail (residues 1–21 of H3) (32, 33, 34) (Fig. 3). Remarkably, binding of the RACK7 PHD finger to oncohistone (K_d_ of 6 μM) is as strong as binding of a typical PHD finger to the unmodified or methylated H3 tail (K_d_ of 1–20 μM) (1, 32). The NMR structure of the RACK7 PHD finger in complex with H3G34R peptide provides mechanistic insights into the recognition of the oncohistone (32) (Fig. 3*A*). Although there is no structure of the RACK7 PHD finger bound to the histone H3 tail available, the recent Lan *et al.* (32), study shows that this domain has separate binding sites for H3G34R and the H3 tail, as different sets of resonances in NMR spectra of the RACK7 PHD finger are perturbed upon the association with each ligand. The most notable characteristic feature of the H3G34R-binding mechanism is that the oncopeptide is oriented almost perpendicular to the β-sheet of the PHD finger, in contrast to the H3 tail that typically lays parallel to the β-sheet (Fig. 3*B*). The major driving force of the interaction with H3G34R is the formation of hydrogen bonds and salt bridges between the positively charged guanidino group of R34 of the oncohistone and the negatively charged carboxyl groups of two aspartate, D104 and D108, of the RACK7 PHD finger (Fig. 3, *A* and *C*). Mutation of D104 abolishes this interaction, and mutation of D108 reduces it by ∼4-fold. In addition, the hydrophobic contacts involving V35 of H3G34R and L121 of the PHD finger contribute to the binding, as mutation of L121 disrupts the complex formation.

Overlay of the structures of the RACK7 PHD finger in complex with H3G34R peptide and the RACK7 PHD-BD-PWWP (PHD-bromodomain-Pro-Trp-Trp-Pro) cassette in the apo state shows that the oncohistone is bound on the side of the PHD finger opposite to BD and PWWP (Fig. 3*C*), therefore the presence of BD and PWWP in the context of full-length RACK7 should not significantly alter binding to H3G34R. However, steric hindrance would preclude binding of the RACK7 PHD finger to both H3 tail and H3G34R concurrently (Fig. 3*D*). We note that ZMYND11, which also contains the multiple readers cassette PHD-BD-(Zn-knuckle)-PWWP, recognizes H3K36me3 through the BD-PWWP interface (35) (Fig. 3*E*). It will be interesting in future studies to assess whether the ZMYND11 PHD finger is able to bind H3G34R, because it contains a set of aspartate and glutamate residues in the same positions as D104 and D108 in RACK7 as well as leucine in the same position as L121 in RACK7.

## Impact of binding to H3G34R

In cells containing nononcogenic histone H3, RACK7 localizes to enhancers through binding of its PHD-BD cassette to H3K14ac and H3K4me1 (34, 36). RACK7 controls enhancer activity by recruiting the H3K4me3-specific demethylase KDM5C to remove H3K4me3 and maintain H3K4me1 (36). Loss of RACK7 increases expression of several known oncogenes and promotes cancer phenotypes in cells. Notably, the PHD finger of RACK7 is the first reader reported to directly interact with H3G34R oncohistone found in pediatric gliomas. This interaction can recruit RACK7 to new genomic locations where it can repress the transcription of tumor suppression–associated genes, including human leukocyte antigens and the major histocompatibility complex class II components (33). Another possibility is that relocating RACK7 away from endogenous targets to the H3G34R sites may lead to overexpression of oncogenic pathway components normally suppressed by RACK7 that contribute to pediatric gliomas.

## Recognition of H3K14ac by DPF

**Figure 4:**
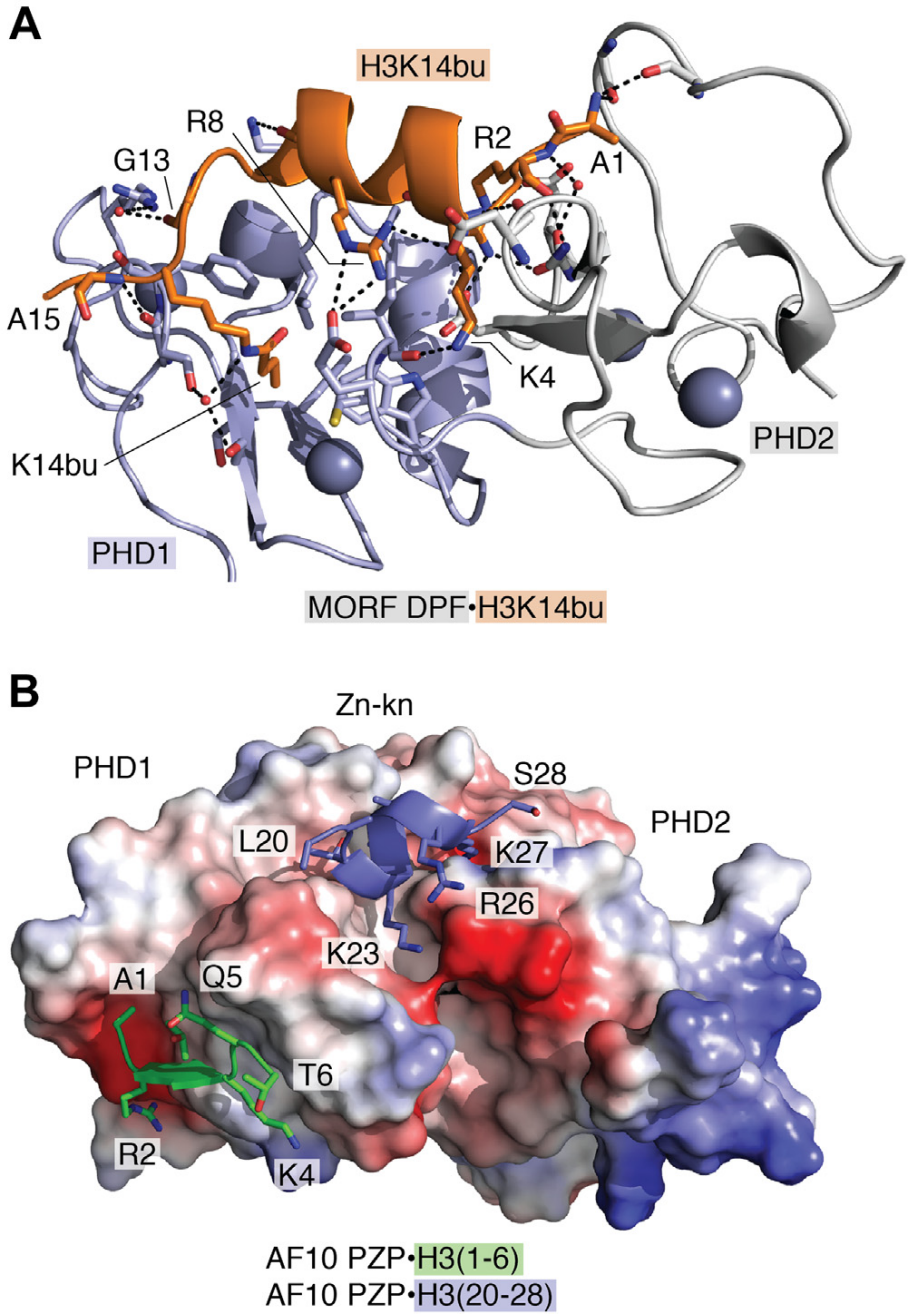
**Structural basis for the recognition of H3K14acyl by DPF and of H3 by PZP.***A*, the crystal structure of the MORF DPF domain in complex with H3K14bu (PDB ID: 5U2J). DPF is shown in ribbon diagram with PHD1 and PHD2 colored *blue* and *gray*, respectively, and H3K14bu peptide is colored *orange*. The histone peptide residues are labeled. *Dashed lines* indicate hydrogen bonds. *B*, overlay of the structures of the H3(1–6)-bound AF10 PZP (PDB ID 7MJU) and H3(20–28)-bound AF10 PZP (PDB ID 5DAH). Electrostatic surface potential of AF10 PZP (shown only for PDB ID 7MJU), with *blue* and *red* colors representing positive and negative charges, respectively. The histone regions H3(1–6) and H3(20–28) are *green* and *blue*, respectively. DPF, double PHD finger; PHD, plant homeodomain.

Originally, the double PHD finger (DPF) of DPF3b and then DPFs of MOZ, MORF, and DPF2 were shown to recognize acylated K14 of histone H3 (H3K14acyl) (22, 37, 38, 39, 40, 41). NMR and crystal structures of the H3K14acyl- (acetyl, propionyl, butyryl, and crotonyl) bound DPFs from DPF3b, MOZ, and MORF provide insight into the selectivity of this reader toward acyl-lysine substrates (22, 38, 40, 41, 42, 43, 44, 45) (Fig. 4*A*). In the complex, the tandem PHD fingers (PHD1 and PHD2) adopt a bean-shaped scaffold, and both are involved in the interaction with H3K14acyl. The residues A1-T3 of H3K14acyl lay in the acidic groove of PHD2 and are engaged in intermolecular electrostatic and hydrogen bonding contacts that are also observed in the complexes of a typical PHD finger and unmodified H3, as described above. The residues K4-T11 of the H3K14ac peptide however adopt an α-helical conformation and interact with the PHD1 finger. The entire side chain of K14acyl inserts deeply into a binding pocket formed by hydrophobic residues of PHD1, indicating that the main driving force of recognition of acyl-lysine by DPFs is hydrophobic in nature. Yet, electrostatic interactions play an important role in the formation of the complex, as DPFs are also capable of associating with unmodified H3 tail. Indeed, multiple hydrogen bonds and salt bridges restrain the side chains of essentially all positively charged residues in the histone tail, including R2 and K4. As a result of such stringent coordination, methylation of either R2 or K4 disrupts binding to H3K14acyl.

## Biological role of the DPF–H3K14acyl interaction

Although acylation enhances the histone binding of DPFs ∼2 to 10 fold, this modest increase in binding affinity has a significant impact on functions of the DPF-containing proteins. DPF3b and DPF2 are components of the BAF chromatin remodeling complexes. Recognition of H3K14ac by DPF3b DPF plays an important role in transcriptional activation of DPF3b/BAF target genes, which is critical for the heart and skeletal muscle development (22, 37). Mutations of the H3K14acyl-binding residues in DPF of DPF2 have been associated with neurological disorders, including the Coffin-Siris syndrome (46). MOZ and MORF are catalytic subunits of the MOZ/MORF acetyltransferase complexes that acetylate H3K23 (43, 47). These complexes regulate expression of the *HOX* genes and are required for development, hematopoiesis, and skeletogenesis (48). Aberrant catalytic activities of MOZ/MORF caused by chromosomal translocations and mutations are linked to aggressive forms of leukemias and developmental diseases. Binding of MOZ/MORF DPFs to acylated H3K14 promotes acetylation of H3K23 by the native complexes and stabilizes these complexes at target gene promoters to activate transcription (43, 47).

## Recognition of H3(1–6) and H3(20–28) by PZP

Two PHD fingers linked by a single zinc finger form an integrated module, the PZP domain (PHD-Zn-knuckle-PHD). Structural studies of PZPs from BRPF1, AF10, and PHF14 reveal a very similar saddle-like globular fold of this domain, which is stabilized by five zinc-binding clusters (49, 50, 51, 52, 53), and all PZPs have been shown to bind to unmodified histone H3 tail, but not identically. While the first PHD finger (PHD1) of PZP from BRPF1, AF10, and PHF14 interacts with the N-terminal region of H3 (residues A1-T6) in a manner similar to a typical PHD finger, PZPs of AF10 and PHF14 also recognize the middle region of histone H3 tail (residues L20-S28) (Fig. 4*B*). This region folds into an α-helix and is bound in a deep channel at the interface of PHD1, Zn knuckle, and PHD2 of AF10 PZP (49). Several intermolecular hydrogen bonds restrain the histone residues T22-K27. The side chain of K27 is particularly fixed by hydrogen bonds formed with three backbone carbonyls of PZP and therefore is not amenable to posttranslational modifications: even mono-methylation of H3K27 disrupts this interaction (49). It is plausible to suggest that since PZPs of AF10 and PHF14 engage both regions of H3 tail concurrently, other PTMs in the H3 tail could modulate this interaction.

## Biological importance of the PZP–H3 interaction

In-depth biochemical and *in vivo* analyses of PZPs underscore the significance of the interactions of this domain for proper functions of the PZP-containing proteins, including BRPF1/2/3, AF10/AF17, JADE1/2/3, and PHF14. AF10/A17 is a cofactor of the H3K79 methyltransferase DOT1L, and binding of AF10/AF17 PZP to H3 is required for H3K79 dimethylation and spreading of the H3K79me2 mark (49, 52). It has been shown that PZP of AF10 engages the nucleosome through multivalent interactions with the entire H3 tail as well as DNA and that incorporation of functional PZP in leukemogenic CALM-AF10 fusions blocks the transforming activity *in vitro* and *in vivo* and abolishes CALM-AF10–driven leukemogenesis *in vivo* (52). Functional PZP of BRPF1 is necessary for the recruitment of the acetyltransferase MOZ/MORF complexes to chromatin and enzymatic functions of these complexes (50, 54). The bivalent interaction of BRPF1 PZP with H3 and DNA also affects the nucleosome dynamics, shifting the DNA unwrapping-rewrapping equilibrium toward the unwrapped state and increasing DNA accessibility (50). JADE1 PZP has been shown to play a role in acetyltransferase activity of the HBO1 complexes and in mediating transcription and cell proliferation (55).

## Strategies to inhibit PHD fingers

**Figure 5:**
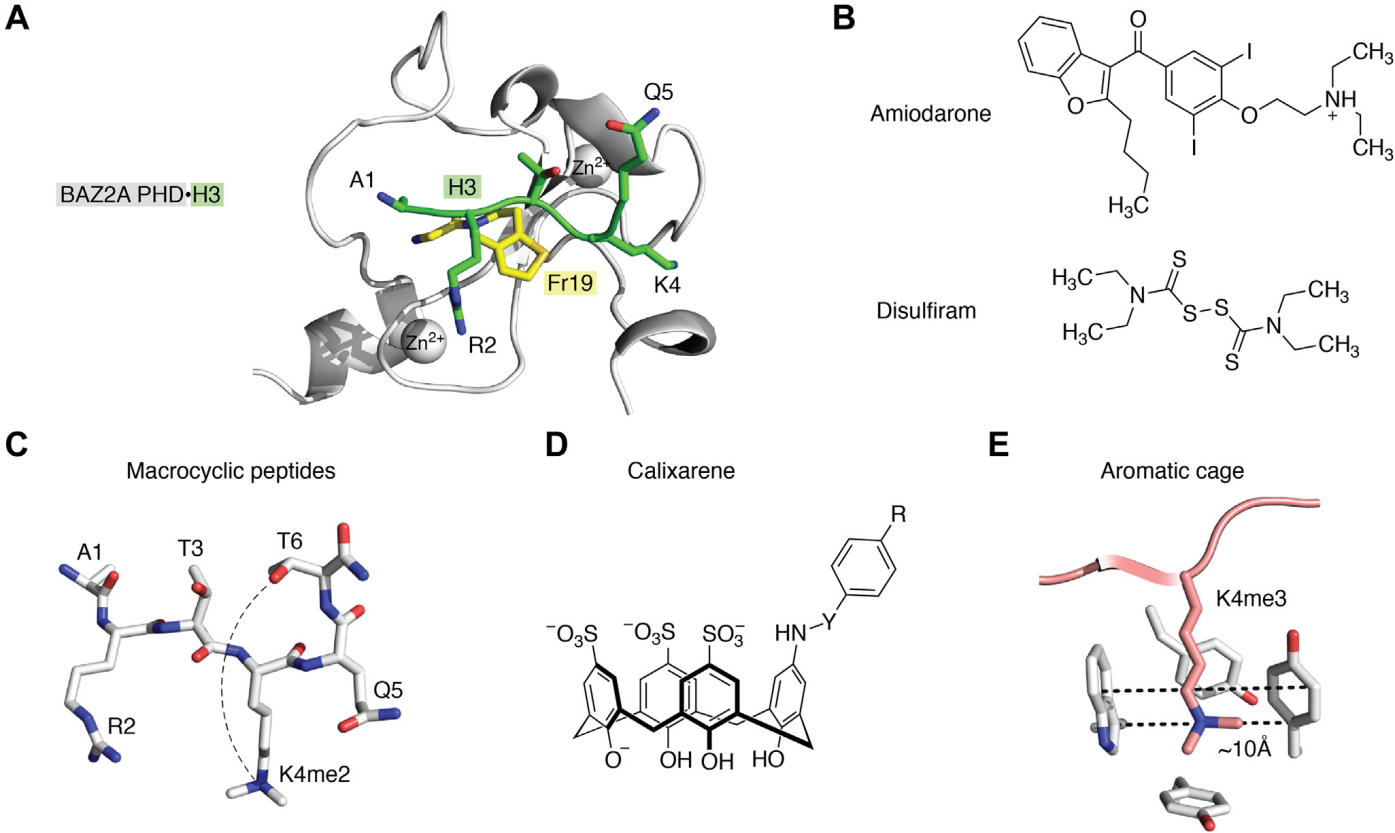
**Blocking histone-binding activities of PHD fingers.***A*, the crystal structure of the BAZ2 PHD finger (*gray*) in complex with H3 peptide (*green*) (PDB ID 6FI0) superimposed with the crystal structure of the BAZ2 PHD finger (not shown) in complex with Fr19 compound (*yellow*) (PDB ID 4Q6F). The H3 residues are shown as *green sticks* and labeled, and the zinc ions are *gray spheres*. *B*, chemical structures of amiodarone and disulfiram. *C*, schematic of the H3-based macrocyclic ligands for PHD fingers. The *dash line* represents a covalent linker between K4me2 and T6 of H3. *D*, chemical structure of calixarene derivatives. *E*, distances between the aromatic side chains in the K4me3-binding cage of the BPTF PHD finger (*gray*). The bound H3K4me3 peptide is *dark pink*. PHD, plant homeodomain.

Because impaired functions of PHD finger–containing proteins have been linked to various human diseases (13, 15), great effort was put forth in the past few years to develop small molecule inhibitors and peptidomimetics to modulate histone-binding activities of PHD fingers. A fragment-based screening approach was successfully used to develop aromatic and heterocyclic small organic compounds that occupy the histone-binding pocket of PHD fingers of BAZ2A and BAZ2B (56) (Fig. 5*A*). Likewise, an NMR fragment–based screening led to the discovery of benzothiazole derivatives that disrupt binding of the Pygo PHD finger to H3K4me2 (57). A HaloTag technology identified inhibitors, including amiodarone and disulfiram, for the third PHD (PHD3) finger of the lysine demethylase 5A (KDM5A) (58) (Fig. 5*B*). A unique strategy to design peptidomimetic antagonists for the KDM5A PHD3 has been developed by Zhang *et al.* (59). These macrocyclic peptides mimic methylated H3K4 but have K4me2 and T6 covalently linked by lactam, thioether, or triazole linkers (Fig. 5*C*). Furthermore, some H3K4me3 mimetics bearing arylsulfonyl fluoride and arylfluorosulfate covalent warheads at Q5 can pull down exogenously expressed KDM5A PHD3 from the lysate of HEK293T cells (59). Alternatively, the H3K4me3–PHD complex formation can be blocked by supramolecular caging compounds and chelating macrocycles, such as calixarenes (60). Calix[4]arenes contain four linked phenol repeats that form a rigid bucket-shape scaffold (Fig. 5*D*). The bucket can trap methyllysine in the internal cavity as its internal volume, and the diameter matches the internal volume and distances within the aromatic cage of PHD fingers (Fig. 5*E*). Water-soluble derivatives of calix[4]arenes were shown to disrupt the association of PHD fingers of cancer drivers, such as ING2, MLL1, MLL5, and KDM5A, with methylated histone H3K4 tail to a varying degree, which depends on binding affinities and methylation state of the ligand (61).

The discovery that RACK7 binds to the H3G34R oncohistone and relocates to new genomic loci associated with H3G34R suggests a novel strategy to develop anticancer therapeutics that target the gain-of-function binding but do not disrupt normal function. Future work is necessary to determine if other chromatin-binding proteins can be competitively redistributed in response to inhibition of the interaction of their PHD fingers (62) with endogenous binding partners. In-depth studies are also needed to better understand how widespread the recognition of atypical histones by PHD fingers is and to establish the determinants of histone-binding specificities.

## Conflict of interest

The author declares that they have no conflicts of interest with the contents of this article.
